# Femtosecond Laser Engraving of Deep Patterns in Steel and Sapphire

**DOI:** 10.3390/mi12070804

**Published:** 2021-07-07

**Authors:** David Pallarés-Aldeiturriaga, Pierre Claudel, Julien Granier, Julien Travers, Lionel Guillermin, Marc-Olivier Flaissier, Patrick Beaure d’Augeres, Xxx Sedao

**Affiliations:** 1 Hubert Curien Laboratory, University of Lyon, Jean Monnet University, UMR 5516 CNRS, F-42000 Saint-Etienne, France; xxx.sedao@univ-st-etienne.fr; 2 GIE Manutech-USD, 42000 Saint-Etienne, France; pierre.claudel@manutech-usd.fr (P.C.); julien.granier@manutech-usd.fr (J.G.); 3 Gravotech Marking, 466 Rue des Mercières, 69140 Rillieux-la-Pape, France; julien.travers@gravotech.com (J.T.); lionel.guillermin@gravotech.com (L.G.); 4 Fibercryst S.A.S., 31 rue Wilson, 69150 Decines Charpieu, France; mo.flaissier@fibercryst.com (M.-O.F.); p.beauredaugeres@fibercryst.com (P.B.d.)

**Keywords:** raster scanning, steel, sapphire, laser engraving, femtosecond laser

## Abstract

Femtosecond laser engraving offers appealing advantages compared to regular laser engraving such as higher precision and versatility. In particular, the inscription of deep patterns exhibits an increasing interest in industry. In this work, an optimization protocol based on constraining overlap ratio and scan number is demonstrated. The proposed method allows changing overlap ratio while maintaining depth in the same range, which reduces the sampling number. This study WAS applied to stainless steel 316 L and sapphire for engravings deeper than 100 μm. Results exhibit overall depths higher than threshold values and allowed to determine optimized engraving quality, for instance, roughness in steel can be reduced while maintaining depth and taper angle by reducing overlap ratio. The optimized laser parameters such as roughness and taper angle factors for sapphire were also found to be as follows: 200 kHz, 86% overlap and 12 J/cm${}^{2}$. As a demonstration, a logo engraving is illustrated at the end.

## 1. Introduction

Laser engraving is a well established technique employed in a vast amount of materials and offering great engraving quality. The most common advantages are: contactless nature, precision, repeatability, productivity and efficiency as well as flexibility and economy [1,2,3]. It can be applied to many fields, especially industry where can be used for: creating molds and dies, engraving for medical applications, custom image patterning beneath a solid surface or engraving of flexographic plates [1]. The most common lasers employed for such purposes are Nd:Yag and CO${}_{2}$ lasers [4]. However, with the advent of ultrafast lasers whose average power has increased during recent decades, they became adequate competitors to the former two types of lasers. The outstandingly short pulse duration of these lasers allows them to perform ablation by direct vaporization through non linear interaction. This kind of interactions are heavily confined within the focal volume and their short duration hinders heat transmission. Thus, they exhibit superior engraving quality, and can adapt to a vast variety of different materials, especially metals and glasses (materials are often used in high-end time pieces and jewelleries). A good number of excellent studies have been gathered and made available to public over the last one or two decades [5,6,7,8,9,10,11,12]. The influences of laser fluence, scan parameters, laser repetition rates and the like on ultrafast laser machining were investigated.

However, there are several key applications in luxury goods industry where the standard engraving depth must be higher than 100 µm, as well as near-vertical cut and smooth engraving floor be highly desirable. The number of relevant publications taking into account of above-mentioned issues is dramatically reduced, and even then, the left ones deal with only one or several of the aspects. For example, roughness, most commonly looked at in many studies has been more often investigated qualitatively [9,11,13,14] rather than quantitatively [2,8,15]. Taper angles were mentioned but their evolution with laser process parameters is rarely discussed [13]. As a consequence, there is only a limited number of femtosecond laser engraving optimization studies and they do not address taper angle, perform limited PRR variation and subject matter mainly covers metal [16,17]. Moreover, the parameter study is limited to raster scanning of simple surfaces (such as rectangles or squares) but not investigated in more complicated shapes such as personalized motifs. For this reason, a new protocol for deep femtosecond engraving inscription addressing these lacks and to meet this industrial demand is presented. It allows different overlap ratio (OL) studies (proportional to writing speed) without significantly reducing engrave depth of 100 μm which reduces the number of samples needed. The key point stems from the scan number, as several scans are usually required for achieving 100 μm depth, constraining this variable to OL though the processing time, allows for a relatively stable lower limit.

This protocol has been employed in two materials: stainless steel 316 L and sapphire, one of metallic nature and the other wide band gap transparent material (8.8 eV). The roughness and taper angle are studied when varying OL, fluence and Pulse Repetition Rate (PRR). Steel as mentioned above, is a comprehensively studied material with some guides of deep engraving but lacks studies combining taper angle and roughness [13,15]. Sapphire is challenging material when high aspect ratio patterns are desired as its low thermal conductivity and a high linear thermal expansion make relatively easy to generate microcracks [18]. Both materials are to be studied in function of scan number alone to determine their behavior and then the constrain are to be applied. In this way, behavior with OL, PRR and high fluence are to be studied. The reason for studying only fluences well above fluence threshold, is that even if there are several studies that prove its low power efficiency [19], it stills remove material at higher time rate which is critical given the depth of the desired inscriptions. Finally, the methodology will also be applied in a logo engraving to demonstrate its final quality.

## 2. Setup and Methodology

**Figure 1:**
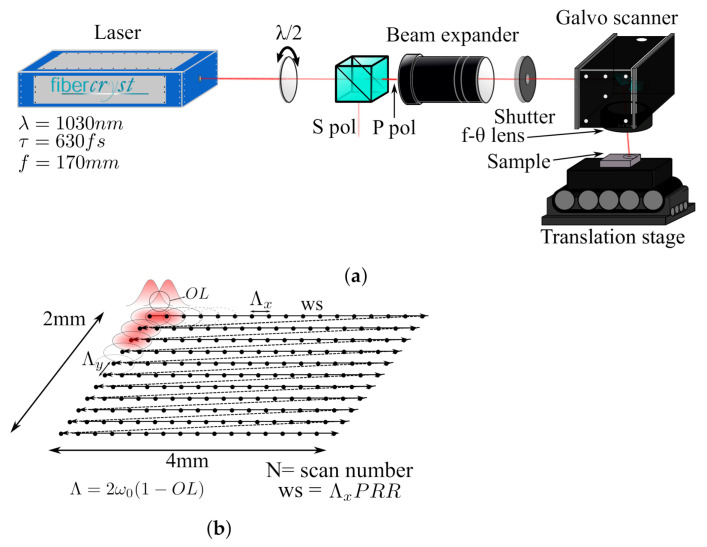
Setup scheme: adjusted beam arrives galvo scanner with a f-*θ* lens (f = 170 mm) that focused on the sample surface which is placed on a XYZ movable stage (**a**). Pattern inscription: pulses are delivered at same direction with an spacing Λ*_x_* = Λ*_y_* (**b**).

In this work, the setup is depicted in Figure 1a, here a laser FEMTO30 from Fibercryst SAS with λ = 1030 nm τ = 650 fs similar to the one previously described in [20,21] has been employed. The laser is optimized to deliver a maximum of 150 μJ/pulse at 200 kHz. The laser beam incides light to a λ/2 retarder and a polarizer beamsplitter for power control, then light passes through a beam expander to adequate laser diameter to the galvanometer scan. This controls the beam position delivered to the f-θ lens with f = 170 mm that focuses the beam on the sample. The beam diameter at focal point can be controlled by changing the magnification power of the beam expander. The reason for installing a tunable beam expander is to meet various industrial processing requirements. The beam diameter $2\omega_{0}$ ($1/e^{2}$ rule) was measured using Gentec-EO camera *Beamage-4M* with a magnifying system, and evaluated within Gentec software *Beamage V1.03*. In this way, the diameter was determined to be 32 and 42 μm for sapphire and steel respectively. For a more precise control, the sample is placed on a XYZ movable stage from Aerotech. This stage is employed to find the focal spot by inscribing an array of drilled holes (50 pulses each) at different heights and selecting the one with lower hole diameter and eccentricity.

By rotating the internal mirrors inside the galvo scanner, several pulses are deposited with a $\Lambda_{x}=\Lambda_{y}$ spacing through numerous X direction scans, forming a 2 × 4 mm rectangle (as depicted in Figure 1b). The relatively large area of inscrption is to ensure a meaningfull roughness measurement. Once the inscriptions are finished, they are cleaned in an ultrasonic bath with water at room temperature.

The studied parameters are the laser fluence F, PRR and pulse overlap OL, which is the surface ratio that two consecutive pulses share (depicted in Figure 1b). As only deep patterns are desired, two protocols were employed. First, for different OLs at same F, the irradiation time has been maintained. Irradiation time is defined as the total amount of time that the laser shutter has been open through the inscription. Unlike inscription time it does not take into account the mirrors displacements to write another lines. The formula of irradiation time is:

(1) $$\Delta t=N\frac{L_{x}L_{y}}{\Lambda^{2}PRR}$$

where $L_{x}$, $L_{y}$ are the rectangle lengths at X and Y direction respectively and Λ is the distance between pulses (assuming $\Lambda_{x}=\Lambda_{y}$, otherwise $\Lambda^{2}=\Lambda_{x}\Lambda_{y}$) and N the number of scans. This value decreases with OL which is proportional to pulse spacing by $\Lambda =2\omega_{0}\left(1-OL\right)$. When adequate OLs are chosen, this change in irradiation time can be compensated by increasing the number of scans. In this way, taking the higher OL of the set, which is 90% in this work, the irradiation time of an inscription N times lower will be simply $\Delta t_{N}=\Delta t_{90}/N$, substituting in Equation (1), relation between the higher OL spacing $\Lambda_{90}$ and the N proportional spacing is:

(2) $$\Lambda_{N}=\sqrt{N}\Lambda_{90}$$

In this way, once the appropriate new pulse spacing $\Lambda_{N}$ has been chosen, it has to be scanned N times to preserve the irradiation time of the highest OL. Table 1 shows a table with OL and Λ employed for both experiments with their respective modifier. This method allows inscriptions at lower OLs without a significant depth reduction.

**Figure 2:**
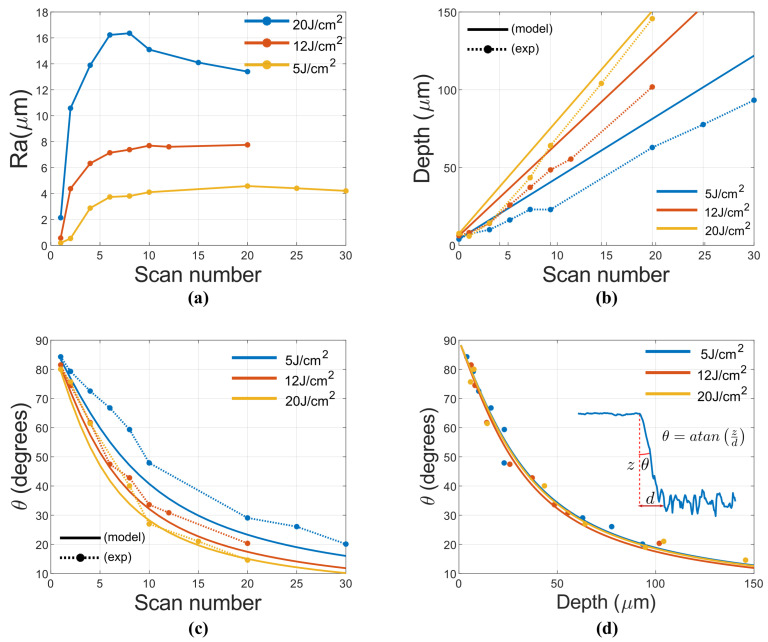
Evolution of different parameter with scan number in steel: Roughness (**a**), model and experimental values of depth (**b**), model and experimental values of taper angle (**c**), behavior of taper angle with depth (z) (**d**).

The second step of the methodology involves finding the adequate number of scan for the highest OL and F. In majority of the cases, one single scan is not enough to achieve high depth even at high OL. Thus, a quick preliminary study for each fluence at an intermediate/low PRR of the chosen set (100 kHz in this work) must be carried out to determine the minimum scan number required to achieve 100±10μm depth. This number will be employed in the procedure with the OL factor discussed above. Once inscriptions have been performed, the engraving quality will be evaluated in terms of depth, taper angle and roughness with a chromatic confocal microscope (Stil, from Altimet). Taper angle is defined as the angle between surface normal and side wall (as shown in Figure 2d).

Stainless steel 316 L and sapphire were purchased from Neyco Vacum and Materials, initial Ra was 0.4 and 0.02 μm respectively. In the case of sapphire, an inscription of logo will be carried out with a lower set of previous parameters. The same setup will be employed, using the same jog speed as writing speed. The logos are to be inspected with both confocal and optical microscope.

## 3. Experimental

### 3.1. Evolution of Depth, Roughness, Taper Angle with Scan Number

Inscriptions of sapphire and steel were performed both at 100 kHz and 90% OL for fluences 512 and 20 J/cm${}^{2}$ (which is maximum fluence available from the experiment setup). Figure 2 depicts roughness (Ra), depth and taper angle (θ) results. Here, Ra behavior with scan number shows a saturation for 6 scans for all the fluence, being saturation value increased with this later. It is noteworthy 20 J/cm${}^{2}$ where roughness reaches a maximum value and then exhibits a slow decay.

In terms of inscription depth, one can try to predict the behavior with fluence employing the following equation [15]:

(3) $$z=\delta_{th}\left(N\right)\ln\left(\frac{F}{F_{th}\left(N\right)}\right)$$

where *z* is the maximum ablated depth, *F* is the current fluence, $F_{th}\left(N\right)$ is the fluence threshold for the number of pulses N employed and finally $\delta_{th}\left(N\right)$ is a fitting parameter linked to thermal parameters such as electronic diffusion and time interaction duration. From this equation, the depth profile can be easily deduced for Gaussian pulses by employing the fluence profile $F\left(r\right)=F_{pk}\exp\left(-2r^{2}/\omega_{0}^{2}\right)$ and substituting in Equation (3):

(4) $$z\left(r\right)=\left\{\begin{array}{cc} \delta_{th}\left(N\right)\left[\ln\left(\frac{F_{pk}}{F_{th}\left(N\right)}\right)-2\frac{r^{2}}{\omega_{0}^{2}}\right] & \delta_{th}\left(N\right)\left[\ln\left(\frac{F_{pk}}{F_{th}\left(N\right)}\right)-2\frac{r^{2}}{\omega_{0}^{2}}\right]>0\\ 0 & \delta_{th}\left(N\right)\left[\ln\left(\frac{F_{pk}}{F_{th}\left(N\right)}\right)-2\frac{r^{2}}{\omega_{0}^{2}}\right]<0 \end{array}\right.$$

From this model, one can predict the result of an engraving by adding all the expected pulses with the spacing $\Delta_{x}$, $\Delta_{y}$ given by the pulse OL:

(5) $$\begin{array}{ccc} z_{G}\left(x,y\right) & = & \delta_{th}\left(N\right)\left[\ln\left(\frac{F_{pk}}{F_{th}\left(N\right)}\right)-2\frac{{\left(x+i\Delta_{x}\right)}^{2}}{\omega_{0}^{2}}-2\frac{{\left(y+k\Delta_{y}\right)}^{2}}{\omega_{0}^{2}}\right]; \end{array}$$

(6) $$\begin{array}{ccc} z(x,y) & = & \sum_{i=0}^{N_{x}}\sum_{k=0}^{N_{y}}z_{G}\left(x,y\right) \end{array}$$

Where $N_{x}$ is the number of pulses per line, $N_{y}$ is the number of lines, *i* and *k* are integers. For 90% OL, the approximate number of pulses per beam diameter is 20, whose threshold has already been experimentally calculated using the well known D-square method [22], being $F_{th\_20\;scans}=0.11$ J/cm${}^{2}$, this agrees well with existing literature [23]. By adjusting Equation (3) to the experimental values, the parameter $\delta_{th}\left(N\right)=14$ nm was found. With this parameter, depth and taper angle were calculated and compared to experimental values as depicted in Figure 2b,c. Here the results slightly deviate for low fluences but for high fluence (20 J/cm${}^{2}$) they agree with the model. Overall, the experimental values exhibit lower depth than theoretic values. This might be attributed to multiscan engraving scheme, where the pre-formed surface structure by earlier scan will influence the subsequent scan. This might lead to less efficiency. As depth increment is linear with the number of scans, it is possible to plot taper angle against depth as depicted in Figure 2d. In this way, an arctan relation is observed. This is explained by a transition region d that is approximately constant with number of scans.

**Figure 3:**
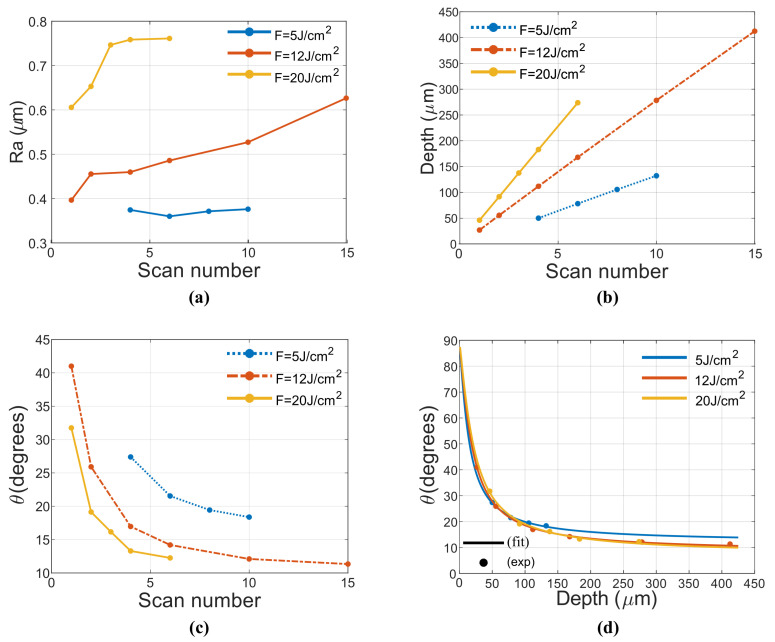
Evolution of diferent parameter with scan number in sapphire: Roughness (**a**), depth (**b**), taper angle (**c**), behavior of taper angle with depth (**d**).

For sapphire, the roughness depends greatly on fluence, as depicted in Figure 3a. For 5 J/cm${}^{2}$, roughness is low (Ra ≈ 0.35 μm) and constant. When it increases to 12 J/cm${}^{2}$, roughness linearly increases and for higher fluences (20 J/cm${}^{2}$) it dramatically increases up to a critical point and then becomes constant. Overall resulting roughness is much lower than steel. It is quite likely that heat accumulation plays a critical role in this behavior.

Behavior of depth and taper angles is more difficult to model as Equation (3) works only for metals. In fact, depth behavior with fluence is linear rather than logarithmic. In terms of depth, behavior is also highly linear as in the metallic model, but for taper angle, this model strongly deviates. In metallic model a transition region approximately constant was assumed. In the case of sapphire it linearly increases with scan number (or depth). The behavior of taper angle with depth is depicted in Figure 3d where it is clear that it fits a $\theta =atan\left(\frac{a+b*dz}{dz}\right)$ and slope b reduces with fluence increment. An explanation to this might be the absorption being sensitive to the incidence angle [24]. In this way, absorption at transition region is reduced compared to engraving surface, thus progressively increasing this later due to the lower ablation efficiency. The taper angle value is in agreement with literature [14].

### 3.2. Engraving Quality Evolution (Depth > 100 μm)

Having carried out an initial parameter study with scan number, the proposed methodology in Section 2 is conducted.

#### 3.2.1. Steel

**Figure 4:**
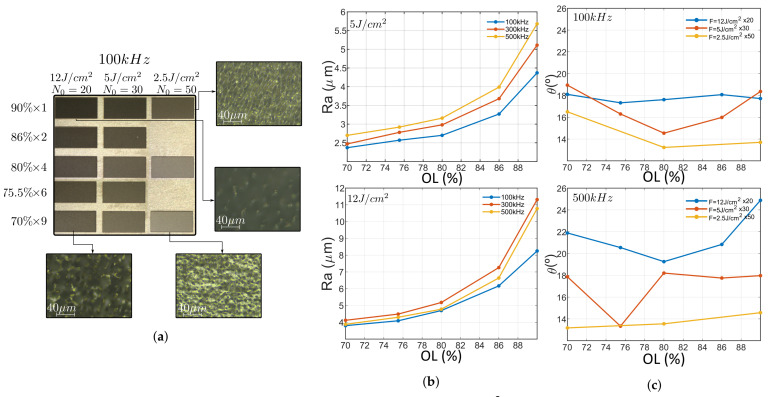
Capture of inscribed rectangles at 100 kHz, fluences 2.5 5 and 12 J/cm^2^ were chosen with an initial scan number (for 90% OL) of 50, 30 and 20 respectively. Each of this fluences were inscribed at 90%, 86%, 80%, 75.5%, 70% OL. Microscope captures shows that high fluence inscriptions exhibit remarkably higher roughness than low fluence inscriptions (**a**). roughness of inscribed rectangles vs. OL for 5 and 12 J/cm^2^ for three different PRRs (**b**). Taper angle vs. OL at 100 and 500 kHz for three different fluences (**c**).

Rectangles were engraved at fluences 2.5, 5 and 12 J/cm${}^{2}$ (requiring 20, 30 and 50 scans to achieve targeted depth, respectively), OLs 90 86 80 76 and 70% for PRRs 100, 300, 500 kHz. Images of inscription results are depicted in Figure 4, it is noteworthy the high number of scans required to reach target depth. This number is far larger than the saturation point discussed in Figure 2a of the previous subsection, which suggest that in this region, Ra is independent of scan number. A careful inspection of inscribed rectangles reveals that gray level change becomes darker with OL and fluence increase. Moreover, high fluence and OL induce low reflectance and rough, oxidized surface as microscope captures at 12 J/cm${}^{2}$ suggest. On the other hand, low fluence and OL induce a smooth surface with higher reflectivity as depicted in microscope capture at 2.5 J/cm${}^{2}$ and 70% OL. This change in ablation quality stems from heat accumulation [9]. In terms of taper angle, values for 100 kHz are more compact that the ones at 500 kHz. In both cases, taper angle increases with F as depicted in Figure 4b. Moreover, at high fluence, the increase of PRR involves an increase of taper angle (and transition region) which is further pronunciated at high OL.

As previously mentioned while studying inscription surface, Ra depends on OL and fluence. Figure 4 depicts Roughness in function of OL at both 5 and 12 J/cm${}^{2}$. Roughness exponentially increases with OL at both fluences with Ra at 12 J/cm${}^{2}$ twice higher than that of 5 J/cm${}^{2}$. This is in good agreement with existing literature [16] but here the behavior of taper angle with fluence is also highlighted. The role of PRR is not that straightforward as the other two parameters. Roughness variation is not remarkable except at high OL. This value, increases with PRR at 5 J/cm${}^{2}$ but when fluence is increased, intermediate PRRs exhibits higher roughness than high PRR.

These observations suggest that for the engraving applications in question, where a smooth floor of the engraved patterns is desirable, small OL should be applied. However, in other cases where a high contrast/black appearance is desirable, high OL could be applied. High PRR and fluence can be used to reduce the over-all process time, this option is thought at a price of compromising roughness Ra and taper angle θ. There is an obvious trade-off between time and quality.

#### 3.2.2. Sapphire

**Figure 5:**
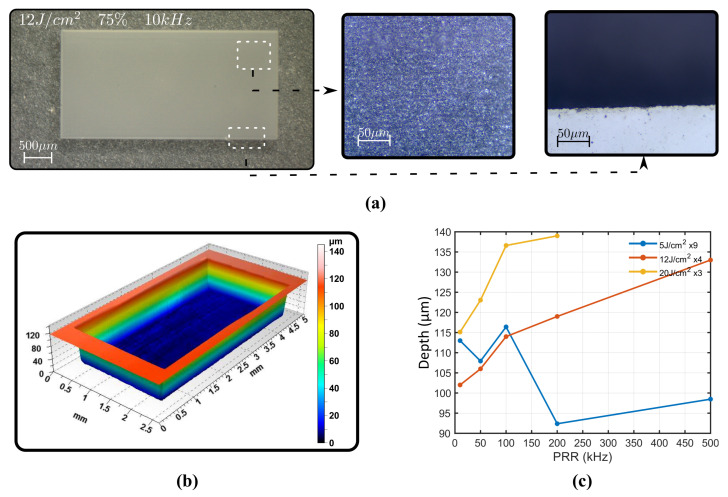
Square inscription at 12 J/cm${}^{2}$, 75% overlap and 10 kHz PRR written 24 times (**a**). Surface profile of that same rectangle with confocal microscope (**b**). Depth of rectangles with repetition rate for different fluences (**c**).

Rectangles were engraved at fluences 5, 12 and 20 J/cm${}^{2}$ (requiring 9, 4 and three scans to achieve targeted depth, respectively), OLs 90, 86, 80 and 76% for PRRs 10, 50, 100, 200 and 500 kHz. One example is depicted in Figure 5a; here microscope images reveal a smooth surface and good quality borders. The surface exhibit a waviness perpendicular to the scanning direction that increases with OL and PRR. The borders reveal no cracks or other defects. This results are consistent with confocal microscope measurements also depicted in Figure 5b. The inscription is deep and homogeneous. A mere 15% of the samples are bellow target depth, being 30% maximum deviation. They correspond to the 5 J/cm${}^{2}$ values where ablation efficiency decreases when PRR increases above 100 kHz. The reason for this behavior could be attributed to this fluences being of the same order of the threshold value, which is $F_{th\_2\;pulses}=2.76$ J/cm${}^{2}$ for two pulses at 100 kHz. This trend is opposite to higher fluences where efficiency increases with PRR. Figure 5c. depicts the depth of inscribed rectangles at 90% OL for studied fluences. For the other values, the efficiency linearly increases (12 J/cm${}^{2}$) or abruptly increases and tends to saturate (20 J/cm${}^{2}$). This trend is also followed at lower overlaps. Moreover, the taper angle is reduced with fluence increment, and also increases for high OL (90%) as depicted in the supplementary material.

**Figure 6:**
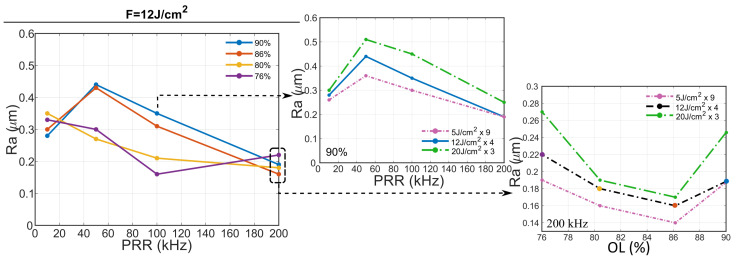
Roughness plot of inscribed rectangles at 12 J/cm${}^{2}$ against PRR at different OLs (**left**). This tendency is consistent at different fluences, as the plot in the middle suggest, where OL is fixed at 90% and the Ra is plotted at different fluences. Low roughness is achieved at 200 kHz for all OL (with 86% a local minimum at all fluences), as shown in the figures on the **right**.

In terms of surface roughness, behavior is strongly dependent with overlap. Here, high OLs (>85%) exhibit a local maximum at 50 kHz while low OLs (<75%) exhibit a local minimum at 100 kHz, intermediate OLs exhibit a monotonic decrement of surface roughness. This tendency is depicted in Figure 6 for 12 J/cm${}^{2}$ but this trend is also accomplished at both lower and higher fluences. As an example, center plot shows 90% OL at 5, 12 and 20 J/cm${}^{2}$. All the fluences exhibit a local maxima at 50 kHz and follow the same trend, being roughness higher at higher fluences, regardless the lower number of inscriptions performed. It is also noteworthy that at 200 kHz, 90 and 75% OLs exhibit higher roughness compared to intermediate values. This trend can be also seen at other fluences as the correct graphs suggest, being 86% the overlap with lowest roughness for the three studied fluences. This trend is also followed by depth and taper angle. Depth exhibits a local maxima at 86% for all repetition rate, being it more pronounced with the increase of PRR. As expected from Section 3.1, the taper angle behaves inversely proportional to the depth, hence exhibiting a local minimum.

### 3.3. Specific Logo Engraving

Once the protocol was carried out, some of the employed parameters were used for engraving a 12 × 2 mm *GRAFEM* logo in sapphire.It is worthwhile to note that here an additional step was taken to make sure a desired laser scan speed can be reached within the logo area (since logo dimensions are small compared to the parametric study of the 4 × 2 mm rectangular areas. The speed limit in confined area should be cross-checked). In this way, overlaps 90% and 76% were employed at both 10 and 100 kHz for all fluences. First, 10 kHz results did not show a remarkable improvement compared to 100 kHz which takes approximately 10 times less to inscribe. This is in good agreement with previous results where low repetition rate brings lower depth and higher taper angle. For this reason, the study is to be focused on the 100 kHz results.

**Figure 7:**
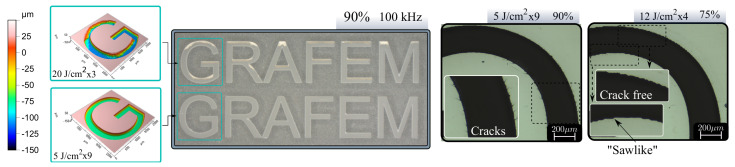
Logo (12 × 2 mm) engraved in sapphire: 20 and 5 J/cm${}^{2}$ at 90% OL and 100 kHz, microscope image (**center**) and confocal surface profile (**left**). Magnified image (×50) of 5 J/cm${}^{2}$ 90% inscription exhibiting microcracks and 12 J/cm${}^{2}$ 75% exhibiting low-overlap effects (**right**).

Figure 7 shows a microscope image of 20 J/cm${}^{2}$ and 5 J/cm${}^{2}$ for 90%. Additional artifacts are appearing in logo engraving, it is clear that higher fluence produce defects, debris and irregular surface as depicted in confocal microscope image. If lower fluences are employed, the result becomes much more smooth but longer process time (274 s while 20 J/cm${}^{2}$ takes 72 s). Moreover, a careful inspection of the engraving reveals micro-cracks at all the fluences for 90% OL. Micro-cracks can appear along specific crystal axis [25] during ultrafast laser machining due to its anisotropic mechanical strength, in our case, the micro-cracks appear to associate to scanning strategy, or OL, the reason this is so has to be further investigated in the near further. Microscope image of Figure 7 (right) shows a magnified part of the G letter for 5 J/cm${}^{2}$. There are no signs of microcracks, but edges exhibit a sawlike profile. This effect is attributed to the high separation between pulses that leaves zones without proper irradiation. This results suggest the use of moderate fluences and overlaps to achieve engravings with best trade off quality.

## 4. Conclusions

A new protocol for deep engraving based on constraining OL and scan number was established. It was applied to stainless steel and sapphire. For stainless steel 316 L, this protocol highlighted the OL decrease as a means of reduce the high Ra induced at High PRR. However, when a strong color contrast is desired, high OL values are more convenient. Moreover, the use of high PRR and fluences can remarkably reduce the over-all inscription time but with an obvious Ra and θ trade-off quality. In the case of sapphire, optimized values for best engraving quality were found at 200 kHz, 86% and 12 J/cm${}^{2}$. More care needs to be given when transferring parametric study results into real pattern inscription practices. Further study in actual inscriptions revealed that high OL values cause microcracks while low ones led to rugged engraving edges. Thus, intermediate OL values suggest best trade-off. In addition, a study of scan number was conducted, obtaining a general trend to both steel and sapphire for depth, taper angle and roughness. It was shown that depth behaves linear with scan number and also exhibits an arctan relation with taper angle where transition region linearly increases with depth in sapphire. This increase is lower at high fluence which gives better taper angles at same depth.

The proposed method is reliable, saves time and gives valuable optimization information to determine best parameters for engraving of deep structures in both materials. This method is being applied to engraving studies of other categories of materials also, such as polymer and ceramics. Its overall objective is to find best conditions for laser engravings by optimizing laser parameters as well as modifying laser source characteristics (PRR, pulse energy, etc.).

## Figures and Tables

**Table 1:** Table with different overlaps (and corresponding spacing) for sapphire and steel with the scan number correction N to adjust same irradiation time (c). n is the number of scans required to obtain 100±10μm depth for $\Lambda_{90}$ (90%).

Sapphire ($2\omega_{0}=32\;\mu$m)			Steel ($2\omega_{0}=42\;\mu$m)		
Λ **(** μ **m)**	**OL(%)**	**N**	Λ **(** μ **m)**	**OL(%)**	**N**
3.2	90	1n	4.2	90	1n
4.53	86	2n	5.94	86	2n
6.4	80	4n	8.4	80	4n
7.84	75.5	6n	10.25	75.5	6n
			12.6	70	9n

## Supplementary Materials

The following are available online at https://www.mdpi.com/article/10.3390/mi12070804/s1.
