# Supplementary material for: Femtosecond Laser Engraving of Deep Patterns in Steel and Sapphire
Source: Micromachines (Basel). 2021 Jul 7;12(7):804. doi: 10.3390/mi12070804 (PMC8306761; doi:10.3390/mi12070804)

Steel

100 kHz

500 kHz

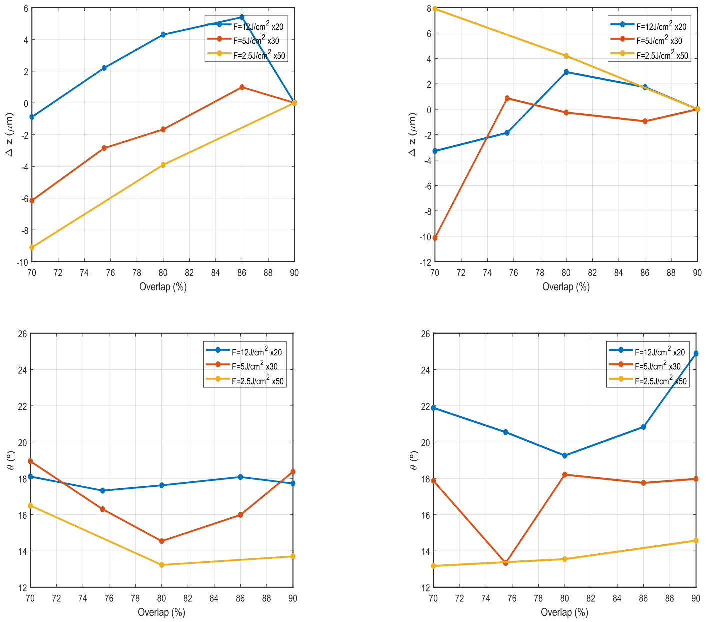

Depth change with overlap: depth at 90% is set as reference to compare its evolution with overlap at different fluences.

Sapphire

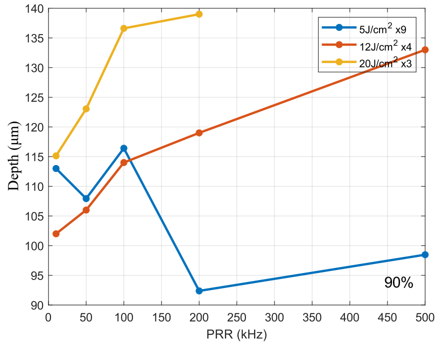

F=20J/cm²

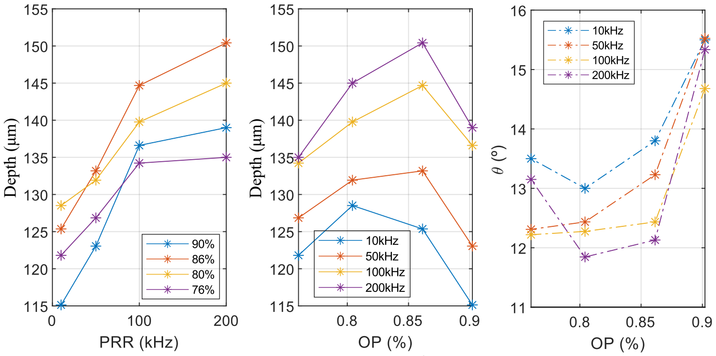

F=12J/cm²

The taper angle is reduced with fluence increase and also increases for high overlaps (90%)

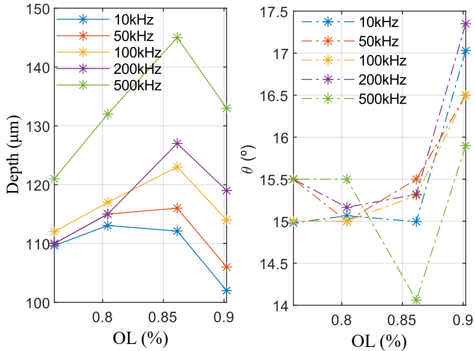

Supplement: Supplementary file 1 [file micromachines-12-00804-s001.zip › micromachines-1237748-supplementary.pdf]
